# Does *Acacia dealbata* express shade tolerance in Mediterranean forest ecosystems of South America?

**DOI:** 10.1002/ece3.1606

**Published:** 2015-07-22

**Authors:** Narciso Aguilera, Carolina Sanhueza, Lubia M Guedes, José Becerra, Sebastián Carrasco, Víctor Hernández

**Affiliations:** 1 Departamento de Botánica, Facultad de Ciencias Naturales y Oceanográficas, Laboratorio de Química de Productos Naturales, Universidad de ConcepciónCasilla 160-C, CP 4030000, Concepción, Chile; 2 Departamento de Botánica, Facultad de Ciencias Naturales y Oceanográficas, Laboratorio de ECOBIOSIS, Universidad de ConcepciónCasilla 160-C, CP 4030000, Concepción, Chile; 3 Facultad de Ciencias Forestales, Laboratorio de Invasiones Biológicas (LIB), Universidad de ConcepciónCP 4030000, Concepción, Chile; 4 Instituto de Ecología y Biodiversidad (IEB)Santiago, Chile

**Keywords:** Early growth, germination, invasion ecology, low light intensity, native forests, non-native forests, non-native range, photosynthetic efficiency

## Abstract

The distribution of *Acacia dealbata* Link (Fabaceae) in its non-native range is associated with disturbed areas. However, the possibility that it can penetrate the native forest during the invasion process cannot be ruled out. This statement is supported by the fact that this species has been experimentally established successfully under the canopy of native forest. Nonetheless, it is unknown whether *A. dealbata* can express shade tolerance traits to help increase its invasive potential. We investigated the shade tolerance of *A. dealbata* under the canopy of two native forests and one non-native for three consecutive years, as well as its early growth and photosynthetic performance at low light intensities (9, 30, and 70 *μ*mol m^−2^sec^−1^) under controlled conditions. We found many *A. dealbata* plants surviving and growing under the canopy of native and non-native forests. The number of plants of this invasive species remained almost constant under the canopy of native forests during the years of study. However, the largest number of *A. dealbata* plants was found under the canopy of non-native forest. In every case, the distribution pattern varied with a highest density of plants in forest edges decreasing progressively toward the inside. Germination and early growth of *A. dealbata* were slow but successful at three low light intensities tested under controlled conditions. For all tested light regimes, we observed that in this species, most of the energy was dissipated by photochemical processes, in accordance with the high photosynthetic rates that this plant showed, despite the really low light intensities under which it was grown. Our study reveals that *A. dealbata* expressed shade tolerance traits under the canopy of native and non-native forests. This behavior is supported by the efficient photosynthetic performance that *A. dealbata* showed at low light intensities. Therefore, these results suggest that Mediterranean forest ecosystems of South America can become progressively invaded by *A. dealbata* and provide a basis for estimating the possible impacts that this invasive species can cause in these ecosystems in a timescale.

## Introduction

The impacts of invasive plants in Mediterranean ecosystems are widely known and vary according to the characteristics of invaders and recipient communities (Fried et al. 2014). One of the most invasive taxa in Mediterranean ecosystems and in the world is the genus *Acacia* (Fabaceae) (Richardson and Rejmánek 2011). According to the global database of Australian acacia records, 386 species have been moved outside Australia by human agency, 71 species are naturalized and 23 are unequivocally invasive (Richardson et al. 2011). The invasion success of *Acacia* species may be related to disturbance events and reproductive traits (Lorenzo et al. 2010). In fact, traits such as ability to effectively reproduce in new locations are common to Australian acacias (Gibson et al. 2011). In addition, the status of *Acacia* invasions may be accelerated under the current global change scenario. Climatic models suggest that about a third of the world's land surface is climatically suitable for Australian acacias (Richardson et al. 2011).

Within this group, *Acacia dealbata* Link (aromo, silver wattle) is a versatile and highly adaptive tree species which has spread all over the world and currently covers a considerable area in the Atlantic climates and some Mediterranean regions: Southern Europe (Lorenzo et al. 2010; Rodríguez-Echeverría et al. 2013; Vazquez de la Cueva 2014), from north western Iberian Peninsula to Italy (Sheppard et al. 2006; Lazzaro et al. 2014), South Africa (Richardson and Rejmánek 2011), and South America (Pauchard and Maheu-Giroux 2007; Fuentes-Ramirez et al. 2011). Specifically, in Chile, it was introduced for ornamental purposes in 1881, (Fuentes et al. 2013) and occupies about 100 000 hectares in the Biobío region (Pauchard and Maheu-Giroux 2007). Some characteristics which make this species a successful invader are its phenotypic plasticity, high capacity for vegetative regeneration from rhizomes and its allelopathic properties (Pholman et al. 2005; Sheppard et al. 2006; Lorenzo et al. 2008).

The spreading of this transformer species has recently received great attention (Richardson et al. 2000); strong impacts to community level of *A. dealbata* invasion have been documented in northwestern Spain and Chile (Lorenzo et al. 2010; Fuentes et al. 2013; Souza-Alonso et al. 2014a; Souza-Alonso et al. 2014b; Souza-Alonso et al. 2015). It enhances nutrient mineralization and decomposition rates (Castro-Díez et al. 2012), modifies the soil microbial communities structure (Lorenzo et al. 2010), decreases diversity and richness of invertebrate communities (Coetzee et al. 2007), and reduces understory plant richness and diversity (Fuentes-Ramirez and Pauchard 2010; González-Muñoz et al. 2012; Lorenzo and Rodríguez-Echeverría 2012). The increased presence of this species in different Mediterranean ecosystems around the world is favored, because it has a high capacity of adaptation to different environmental conditions (Vazquez de la Cueva 2014).

Plant invasions depend highly on disturbance regimes to be successful (Martin et al. 2008; Fuentes-Ramirez et al. 2011). In Chile, generally *A. dealbata* is associated with roads, riparian habitats, and disturbed areas (Fuentes-Ramirez and Pauchard 2010). However, the possibility that it can penetrate the native forest during the invasion process cannot be ruled out. This statement is supported by the high capacity of establishment and growth of *A. dealbata* within the native forest, a behavior that is not observed under its own canopy (Fuentes-Ramirez et al. 2011). Nonetheless, this contrasts with the strong heliophyte character of *A. dealbata* in its native range (Valero and Picos 2009). Also, some field observations suggest that regeneration of this species under its own canopy could be more limited as a result of allelopathic effects, rather than low light intensities (N. Aguilera et al. unpubl. data). Several studies report the phytotoxic effects of *A. dealbata* on native species and soil microorganisms (Lorenzo et al. 2008, 2010, 2011; Lorenzo and Rodríguez-Echeverría 2012; Souza-Alonso et al. 2014a; Souza-Alonso et al. 2014b, 2015; Aguilera et al. 2015), although only Souza-Alonso et al. (2014a) refers to autotoxicity.

From a physiological point of view, the shade tolerance of a given plant is defined as the minimum light under which a plant can survive (Baltzer and Thomas 2007). Although many plants can tolerate low light conditions, only a fraction of them can reproduce under these conditions (Valladares and Niinemets 2008). The most common response of forest invasive species is shade tolerance (Martin and Marks 2006), but the scope of forest invasions by shade-tolerant exotics is unknown (Martin et al. 2008). In this context, it is well documented that forest understories could be more prone to invasion than forest canopies, as most do not appear saturated in terms of either biomass or species diversity (Gilbert and Lechowicz 2005). On the other hand, although the invasion of forests by shade-tolerant exotics may be a slower process than the establishment of exotic species in disturbed or open ecosystems, its long-term effects are likely to be just as pervasive (Martin et al. 2008). Shade-tolerant exotic species are also the most likely to invade protected natural areas in tropical and Mediterranean ecosystems (Gilbert and Lechowicz 2005). A few studies of shade-tolerant exotics indicate that they can have strong impacts on native understory diversity and structure (Martin et al. 2008).

The specific ecophysiological mechanisms by which *A. dealbata* is a successful invader in South America are not well known until now. Then, our hypotheses is that *A. dealbata* in Mediterranean forest ecosystems of South America is shade tolerant, being able to maintain high photosynthetic performance at low light intensities. Therefore, the aims of this study were (1) assessing the ability of *A. dealbata* to establish in the forest under the canopy of native and non-native forests and (2) determining the behavior of germination, early growth, and photosynthetic performance of *A. dealbata* at low light intensities.

## Materials and Methods

### Study area and plant material

**Figure 1:**
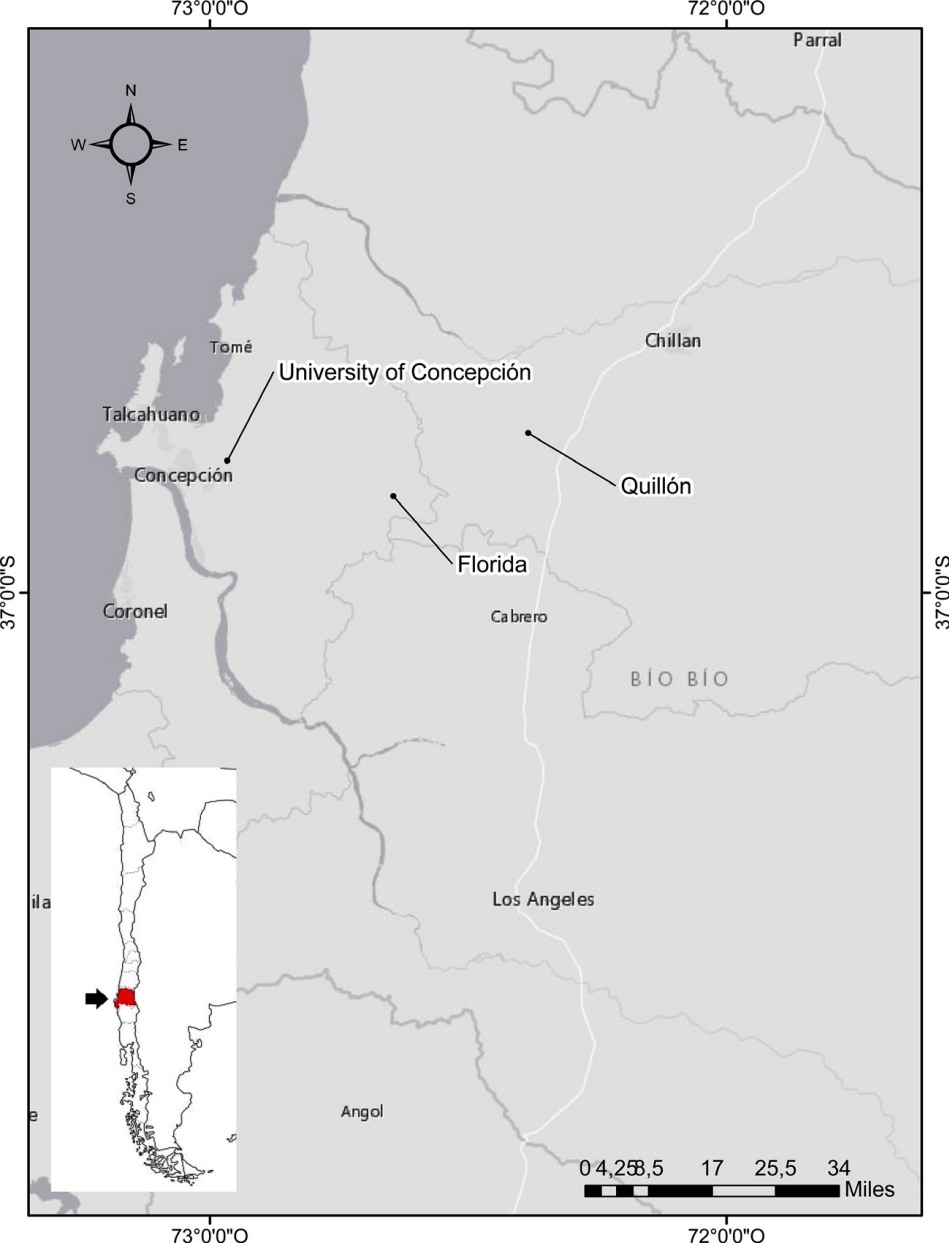
Study sites in Mediterranean forest ecosystems of South America (Biobío region, southcentral Chile).

An exploratory study of the shade tolerance of *A. dealbata* under field conditions was conducted at three sites in the Biobío region, southcentral Chile (Fig. 1), during summer (January–February) for the years 2012, 2013, and 2014. This region of Chile is characterized by Mediterranean climate with average annual temperatures of 12.4°C, relative humidity of 87% and rainfall of 827 mm (Santibáñez and Uribe 1993). The first site of study is located in the Florida commune (36°49′24.69″ S, 72°40′05.91″ W at 256 m.a.s.l), which predominantly has an undulating relief. Often, the landscape is dominated by extensive stands of *A. dealbata*, displacing other species of native and non-native plants. There are also patches of native forests with the presence of *Quillaja saponaria*, *Peumus boldus*, *Lithrea caustica,* and *Cryptocarya alba*. In the understory, monocotyledonous and dicotyledonous herbs are observed. The second site was in the Quillón commune (36°50′58.81″ S, 72^0^32′4.91″ W at 140 m.a.s.l). This area is slightly undulating. The landscape is dominated by shrubs, forest patches with the same species listed above, isolated trees of these native species, and frequent stands of *A*. *dealbata*. The third site is located in campus of the University of Concepción (36°49′42.33″ S, 73°01′54.95″ W at 62 m.a.s.l); specifically in a strongly undulating area. A part of this area was deforested (native trees and shrubs) and reforested with eucalyptus (*Eucalyptus globulus*) for wood and paper industry. The nondeforested part is dominated by native trees belonging to the Nothofagaceae family, along with native trees of *Aristotelia chilensis* and *Drimys winteri*, among others. There are also many small stands of invasive *A*. *dealbata* and *Teline monspessulana* that occupy empty spaces and edges of a path and penetrate progressively into the eucalyptus plantation (non-native forest). These came from 10 different trees in each area and were taken directly from the pods about to expel the seeds. They were stored in closed plastic bags and carried back to the laboratory and refrigerated (∼8°C) for about 2 years until they were used in the experiments.

### Shade tolerance exploration in field conditions

In the study sites, about 1 ha of forest and forest patches were explored in order to quantitate the amount of *A. dealbata* plants growing under the canopy. For this, we covered strips of about 10 m wide, where we they were counted and measured all plants of *A. dealbata*, which allowed us to cover the entire area of exploration. At the same time, we recorded the number of plants of *A. dealbata* found within range of 0–10 m, 10–20 m and over 20 m, starting from the edge of the forest or wooded patches toward the inside of the targeted area. This was performed using a tape measure and establishing a standard equivalent of 1 m by step. Simultaneously, we counted the amount of *A. dealbata* plants that were located at height ranges of 0.1–1.0 m, 1.0–2.0 m and over 2.0 m. Only plants located higher than 0.1 m were considered, because from that point, they have enough vitality and independence to survive more successfully when they come from seeds. This aspect was verified in all cases; as such, we removed enough soil to inspect whether each plant was dependent or not of any root from an adult plant.

### Shade tolerance exploration in controlled conditions

#### Simulation of light intensity under canopy

Light intensities in shaded conditions were evaluated by measurement of light under the canopy of native forest, eucalyptus plantations, and stands of *A. dealbata*. Thirty random light measurements were made under all possible forest scenarios: on cloudy, partly cloudy, and sunny days. The average of light intensity for each environment resulted in 9 *μ*mol photons m^−2^sec^−1^ (cloudy), 30 *μ*mol photons m^−2^sec^−1^ (partly cloudy), and 70 *μ*mol photons m^−2^sec^−1^ (sunny). Light intensities were measured with a LI-190SA quantum sensor (LICOR, Lincon, NE) and were simulated in a growth chamber (Bioref-Pitec, Santiago, Chile) by placing different numbers of compact fluorescent lamps in each of the three sections that divided the camera.

### Bioassay of germination and early growth

A pool was prepared with equal amounts of *A. dealbata* seeds from each of the study sites. Thirty seeds of *A. dealbata* were uniformly placed in Petri dishes (9 cm diameter) lined with a Whatman No 1 paper dis soaked with 5 mL of distilled water. Dishes were sealed with Parafilm® to prevent evaporation: They were randomly placed in a growth chamber at 70% to 75% relative humidity, 12 hours light/dark, and 20 °C. Each light intensity (9, 30 and 70 *μ*mol m^−2^sec^−1^) was a treatment. Ten replicates (Petri dishes) were maintained for each treatment until end of the bioassay. The bioassay lasted 23 days, because after that date, the seeds were softened and showed signs of embryo necrosis. Germination was calculated according to Fernandez et al. (2013), and the value was expressed as percentage (GP). Radicle length (RL) and hypocotyl length (HL) of each seedling were measured and expressed in mm.

The evaluated seedlings were immediately transplanted into small pots (250 cm^3^). The pots were filled with a mixture of soil and organic matter extracted from the first layer (5–7 cm) of the substrate naturally formed under the canopy of native forest located on University of Concepción's campus. The substrate was moistened, and transplant was performed carefully without the roots being damaged. Then, the substrate was pressed so that the roots sufficiently anchored and remained in contact with it. Growing conditions were similar to the bioassay described above. The plants were irrigated weekly without saturating the substrate. The experiment was conducted for 2 months, and at the end of this period, physiological performance was evaluated.

### Gas exchange measurements

Measurements of light response curves were performed at different light intensities (0 to 2000 *μ*mol photons m^−2^ sec^−1^) using an infrared gas analyzer (LI-COR 6400) equipped with an automatic universal leaf cuvette (Li-Cor). The CO_2_ reference concentration was 400 ppm, with a flow rate of 300 mol sec^−1^ and 40–60% external relative humidity. The temperature inside the leaf chamber was maintained at 20°C. Gas exchange values given by Li- Cor 6400 were corrected using the ratio cuvette area/actual leaf area as a correction factor. Measurements were made in five leaves of five different plants selected at random in each treatment.

### Fluorescence measurements

Energy partitioning at PSII level was assessed by fluorescence chlorophyll *a* analyses using a pulse-amplitude fluorimeter (FMS II, Hansatech Instruments). According to the terminology of Van Kooten and Snel (1990), minimal fluorescence (Fo) was determined by applying a weak modulated light (0.4 *μ*mol m^−2^ sec^−1^) and maximal fluorescence (Fm) was induced by a short pulse (0.8 sec) of saturating light (9000 *μ*mol m^−2^ sec^−1^). After 10 sec, an actinic light was turned on for 5 min to obtain fluorescence parameters during steady state photosynthesis. Saturating pulses were applied after a steady state of photosynthesis was reached to determine the maximal fluorescence in the light (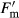) and steady state fluorescence in the light (*F*_s_). Maximum efficiency of PSII in light capture (*F*_v_/*F*_m_), effective quantum yield of PSII (ΦPSII), and electron transport rate (ETR) were calculated in accordance with Genty et al. (1989). The fraction of PSII centers in open state (qL) and nonphotochemical quenching (NPQ) were calculated as reported by Kramer et al. (2004). The fluorescence measurements were performed at photon flux densities of 0 to 1600 *μ*mol photons m^−2^ sec^−1^.

The partitioning of absorbed light energy was estimated according to Hendrickson et al. (2004). The allocation of photons absorbed by the PSII antennae to photosynthetic electron transport and to photochemistry of PSII was estimated as ΦPSII = 1 − (*F*_s_/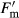) corresponding to the effective quantum yield of the PSII. The fraction of light absorbed by the PSII antennae that is dissipated thermally via ΔpH and/or xanthophyll regulated (NPQ) was calculated as follows: ΦNPQ = (*F*_s_/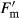) − (*F*_s_/*F*_m_), corresponding to the yield of energy dissipation by downregulation (Song et al. 2010). The light absorbed by the PSII antennae that is lost by either constitutive thermal dissipation (Ff,D) was calculated as follows: ΦFf,D = *F*_s_/*F*_m_ (Cailly et al. 1996). The percentage distribution of the different components of light energy partitioning was established according to the following parameters: PSII, which represents the dissipation by photochemical; NPQ, which represents the nonphotochemical quenching to antenna level indicating which percentage of the energy is lost as heat; and, finally, Ff,D, which represents the constitutive thermal dissipation at the reaction center level. The analysis was performed for every growth light treatment at two light intensities (65 and 1600 photons m^−2^sec^−1^, corresponding to low and high light, respectively).

### Chlorophyll measurements

To estimate chlorophyll content from every growth treatment, we used a handheld Chl meters SPAD-502 (Minolta Camera Co., Osaka, Japan). This has a 0.2 × 0.3 cm^2^ measurement area and calculates an index in “SPAD units” based on absorbance at 650 and 940 nm. The claimed accuracy of the SPAD-502 is ±1.0 SPAD units. Five separate measurements were made on each leaf; we used the arithmetic mean of these measurements for all subsequent analyses.

### Statistical analysis

Bioassays were established on the basis of a completely randomized experimental design. The results of these experiments were subjected to one-way ANOVA (factor: growth light). Before ANOVA analysis, data normality and homogeneity of the variances were, respectively, evaluated with Kolmogorov–Smirnov and Levene tests. Tukey's test was applied for comparison between treatments. Differences between the values were considered significant at *P* ≤ 0.05. All the statistical analyses were performed with STATISTICA v6.0 (Statsoft, Tulsa, OK).

## Results

### Evidences of shade tolerance under field conditions

**Figure 2:**
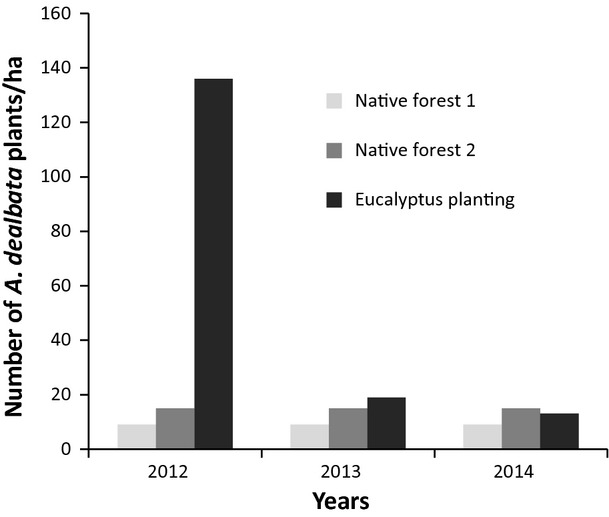
Total of *Acacia dealbata* plants under the canopy of native forest 1 (Florida commune), native forest 2 (Quillón commune), and eucalyptus planting (University of Concepción) for three consecutive years of monitoring.

Systematic monitoring of the study sites during three consecutive years provided robust evidence that indicated the potential shade tolerance of *A. dealbata* in its non-native range. These evidences are based on the amount of *A. dealbata* plants detected that survive under the canopy of native and non-native forests (Fig. 2). It is important to note that the number of such plants remained almost constant under the canopy of native forests during the years of study. However, the number of *A. dealbata* plants decreased by about 85% under the canopy of non-native forest (eucalyptus plantation) during 2013 and 2014 (Fig. 2). The number of *A. dealbata* plants detected under the canopy of the native forest 2 (Quillón commune) was slightly higher than the one found under the canopy of the native forest 1 (commune of Florida) throughout all the years of the evaluation (Fig. 2).

**Figure 3:**
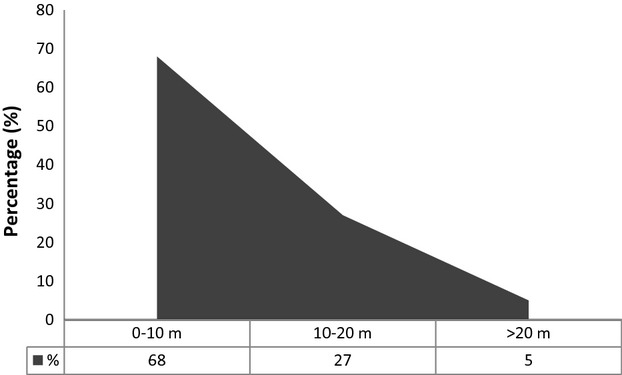
General percentage of plants of *Acacia dealbata* distributed under the canopy at different ranges, from the forest edges inwards.

Moreover, the distribution pattern of *A. dealbata* plants under the canopy varied from the forest edge toward the inside. About 70% of plants were recorded in the first 10 m, decreasing considerably toward the deep forest (Fig. 3). Similarly, almost 80% of the plants did not surpass 1 m of height and less than 10% exceeded 2 m in height (Table 1). Under the canopy of the native forest 1 were recorded more adult trees (estimated by height) of *A. dealbata* that under the canopy of the native forest 2 (Table 1). However, approximately 85% of all *A. dealbata* plants were found under the canopy of the eucalyptus plantation and only 5% of those exceeded 2 m in height.

**Table 1:** Frequency distribution of the number of *Acacia dealbata* plants under forest canopy in the monitoring areas according to three classes established a priori

	Classes			
Monitoring area	0.1–1.0 m	1.0–2.0 m	>2.0 m	Total
Native forest 1	1	3	5	9
Native forest 2	6	7	1	14
Eucalyptus planting	117	12	7	136
Total	124	22	13	159
Percentage (%)^1^	78.0	13.8	8.2	100

1 Percentage of each class in relation to total.

### Effect of light intensities on germination and early growth

**Figure 4:**
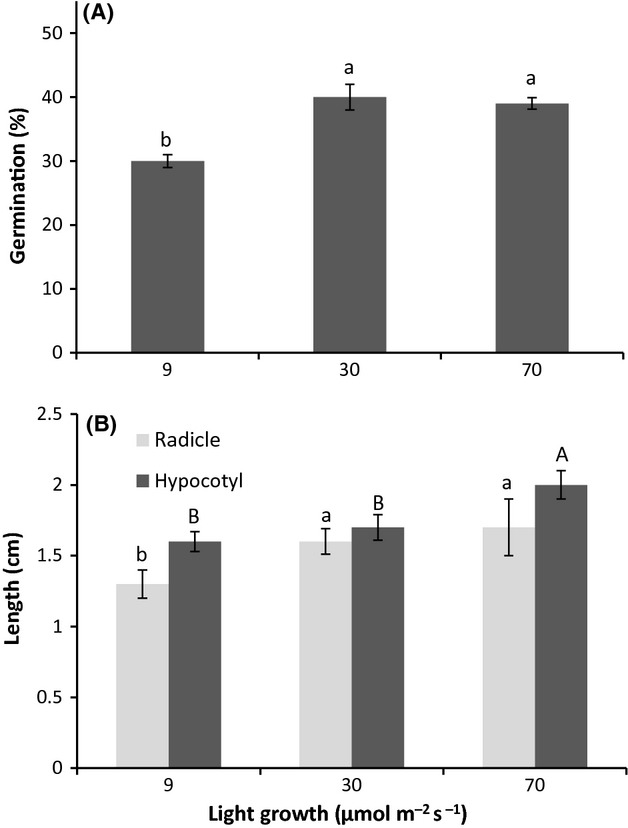
Germination percentage of *Acacia dealbata* (A) and radicle and hypocotyl length (B) in response to growth light intensities. Values shown are mean ± SE. Different letters above bars denote significant differences between treatments after one-way ANOVA (*P* < 0.05) and Tukey's post hoc test. Capital letters represent the variation among radicle length, while lowercase letters represent variation among hypocotyl length (B).

The germination percentages were high in all three levels of light intensities. Nevertheless, the two treatments with higher light intensity surpassed significantly the lowest light intensity treatment as to GP (*P* < 0.001) (Fig. 4A). In all cases, the germination fluctuated approximately between 30% and 40%. The RL behaved similarly, showing statistical significance (*P* < 0.001) for the two higher light intensities compared to the lowest (Fig. 4B). In contrast, the HL under low light intensities was significantly lower (*P* < 0.001) than the one under the highest level of light (Fig. 4B). The growth of *A. dealbata* in its early stages was very slow. In general, the growth behavior which is shown in Fig. 4B indicates that the plants did not exceed 2.3 cm of their total size during the first month of culture.

### Effect of light intensities on photosynthetic parameters

**Figure 5:**
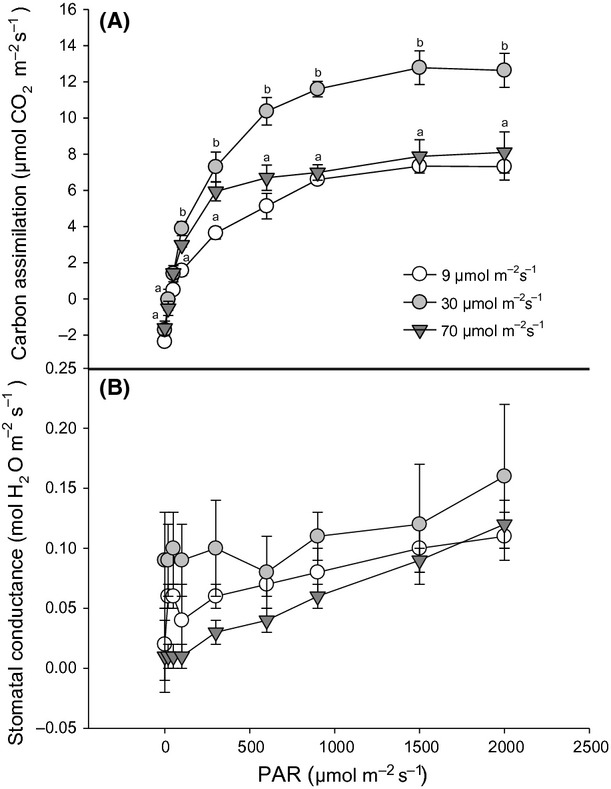
Variation in gas exchange parameters in seedlings of *Acacia dealbata* grown in three light environments and measured at 20°C. Carbon assimilation (*μ*mol CO_2_ m^−2^sec^−1^) represents CO_2_ assimilation measured under different light intensities (A). Stomatal conductance (mol H_2_O m^−2^sec^−1^) indicates stomatal openness under different light intensities (B). Values shown are mean ± SE. Different letters denote significant differences between growth light intensities after one-way ANOVA (*P* < 0.05) and Tukey's post hoc test (A).

**Figure 6:**
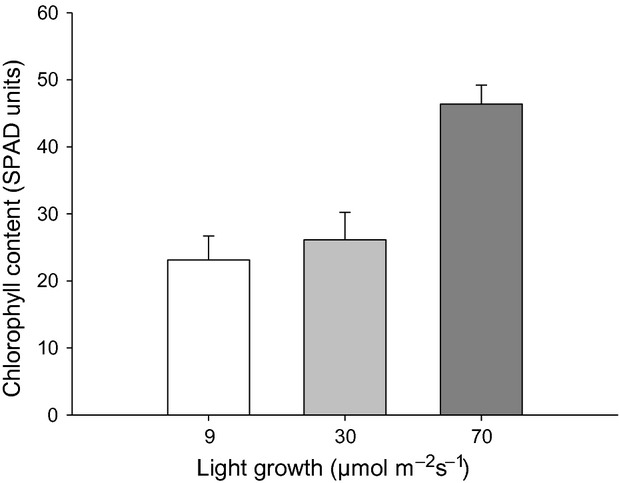
Chlorophyll content in seedling of *Acacia dealbata* grown in three light environments. Amounts of chlorophyll are expressed in “SPAD units” based on the absorbance at 650 and 940 nm. Values shown are mean ± SE. Different letters above bars denote significant differences between treatments after one-way ANOVA (*P* < 0.05) and Tukey's post hoc test.

For individuals of *A. dealbata*, assimilation and light response curves were not increased in plants grown under higher growth light condition (Fig. 5A). Indeed, the maximum photosynthetic rate (12 *μ*mol CO_2_ m^−2^sec^−1^) was shown by plants grown at 30 *μ*mol photons m^−2^sec^−1^, indicating that higher photosynthetic response was not caused by growth light conditions. Stomatal conductance was not significantly different between light treatments (*P* > 0.05). However, stomatal openness increased in response to an increment of light intensity in plants under the light regimes (Fig. 5B) tending to be higher in the medium light treatment (30 *μ*mol photons m^−2^sec^−1^). This could partly explain the highest photosynthetic rate shown in this treatment. The chlorophyll content was significantly higher for individuals grown under the highest light intensity (Fig. 6), although it did not result in a higher photosynthetic rate.

**Figure 7:**
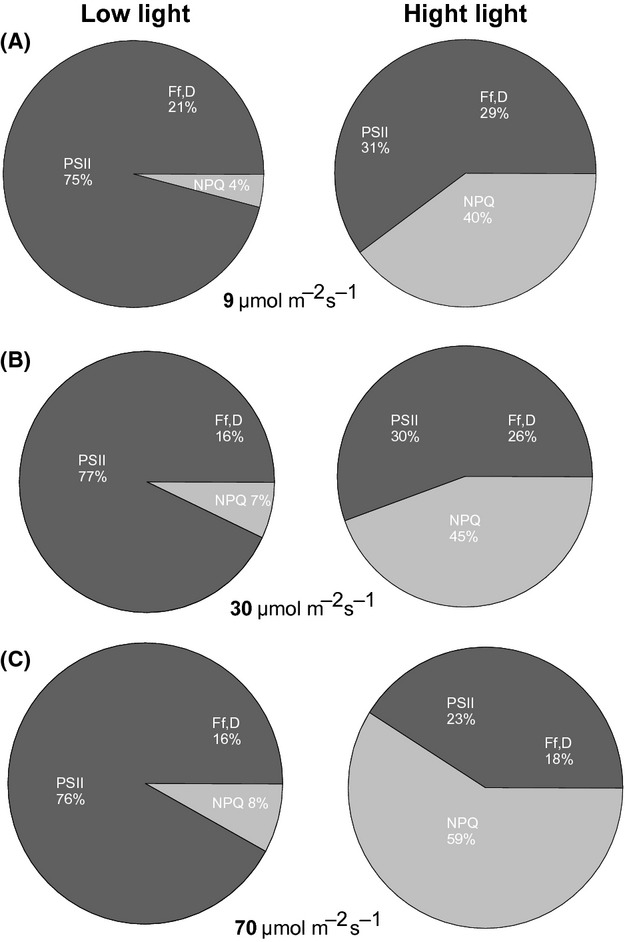
Percentage distribution of the different components of light energy partitioning in seedlings of *Acacia dealbata* grown in three light environments. Low light and high light represent light intensities measurement corresponding to 65 and 1600 *μ*mol m^−2^sec^−1^. PSII represents dissipation by photochemical processes, NPQ represents the non-photochemical quenching, and Ff,D, represents intrinsic dissipation.

Individuals of *A. dealbata* grown under all light regimes showed that at low light, most of the energy is dissipated by photochemical process (Fig. 7A, B, and C left side). This result is accordance with the high photosynthetic rates that this plant showed despite the low intensity under which it was grown (From 9 to 70 *μ*mol m^−2^sec^−1^). The increase in NPQ under higher growth light suggests that this species starts to activate thermal dissipation mechanisms as it gets increasingly exposed to light during its development. At high light measurement (1600 *μ*mol m^−2^sec^−1^) (Fig. 7A, B, C right side), most of the excess light is dissipated by NPQ at the antenna level. Heat dissipation was higher in plants grown under the highest growth light (70 *μ*mol m^−2^sec^−1^), which could be related to a higher pigment concentration (Fig. 6). However, xanthophyll pigments which are involved in heat dissipation were not evaluated in this study.

## Discussion

The absence of sapling individuals of *A. dealbata* under its own canopy made us think, initially, that low light intensity was the cause of the phenomenon (N. Aguilera et al. unpubl. data). However, the number *A*. *dealbata* individuals under the canopy registered in each study areas indicated that this invasive species is able to respond favorably to variations in light intensity in its non-native range. Many studies have shown that when an exotic species arrives in a new environment, its morphology, behavior, and genetic characters may change rapidly, even though leap changes, in order to form the features which are beneficial to its survival and development (Chengxu et al. 2011). Other studies in Europe and Australia have revealed that *Cytisus scoparius* does not recruit beneath dense stands of conspecifics in its native range (Paynter et al. 1998), yet where it is exotic, in Australia, seedlings readily establish under monocultural stands of conspecifics (Sheppard et al. 2000). Pholman et al. (2005) have also shown that widespread species, such as *A. dealbata*, display greater plasticity than restricted-range species in key seedling establishment traits. These authors suggested that *A. dealbata* seedlings show fundamental differences in the physiology and plasticity of leaflets with respect to restricted-range species. These traits would help *A. dealbata* to efficiently utilize diverse habitats in invaded regions (Lorenzo et al. 2010).

Each of the explored forests showed some peculiarity in relation to the presence of this invasive species under their canopy. For example, in the native forest 2 that were observed clearer signs of major disturbance (felled trees) than in the native forest 1, the former showing, therefore, a lower native trees density than the latter. This facilitated greater penetration and establishment of *A. dealbata* propagules in the native forest 2. The uniform behavior of the number of *A. dealbata* plants registered under the canopy of both native forests during the three consecutive years of monitoring indicates that penetration process within the forest is slow. This statement is supported by the fact that less than 10% of *A. dealbata* plants surpassed 2 m in height. In this case, we are assuming that there is a direct relationship between the height and the age of plant (an older age corresponding to the tallest plants). At the same time, this is consistent with the fact that the invasion front presented the greatest abundance of *A. dealbata* plants within the first 10 m of the outer fringe of the forests. This constituted the largest source of propagules that will increasingly occupy new areas within the forest. As such, the greater has been the disturbance of the native forest in its depth, the greater will be the amount of empty niches (Levine and D'Antonio 1999; Mack et al. 2000; Hierro et al. 2005) that can be quickly occupied by these new propagules. This implies that some native assemblages might be open to species that have unique functional attributes (i.e., *A. dealbata*) and can access niche space that is not inhabited by natives (Callaway and Maron 2006).

Besides the presence of *A. dealbata* plants growing under the canopy, the number of roots that have penetrated the native forest and that come from its periphery should also be considered. In this way, the invasive species can start pressing native plants for resources (Hierro et al. 2005). Normally, invasive exotic plants might owe their success to the fact that they have novel functional attributes that enable them to access free resources that are not depleted by natives (Shea and Chesson 2002). In addition, new propagules produced by agamic reproduction shapes will ensure that new plants can establish into the native forest from shoots that develop from their roots. Most of these plants can be associated with a mother plant which survives with relative ease and can be highly competitive (Valero and Picos 2009). Several exotic species, among them *A. dealbata*, can successfully reproduce by way of gamic and agametic reproduction (Richardson and Kluge 2008; Marchante et al. 2010). These features ensure that this invasive species multiply faster in its non-native range (Lorenzo et al. 2010).

Regarding the above, it is important to note that between 50% and 60% of the *A. dealbata* plants that were located in the first 10 m from the edge of the forest come from the roots of adult plants (offshoot). However, from 10 m toward the deep forest, more than 90% of the plants were not connected with the roots of other plants. This indicates that most plants of *A. dealbata* that were found in the deep of native and non-native forest were multiplied from seeds. Probably, the displacement of seeds toward the deep forest was due to runoff caused by rain and ants. This reasoning is consistent with the fact that ninety percent of the world's myrmecochores are found in the Southern Hemisphere (Gómez and Espadaler 1998). Furthermore, it has been informed that *A. dealbata* has been benefited from the dispersion by ants (Edwards and Westoby 1996; Lorenzo et al. 2010).

Moreover, the large number of *A. dealbata* plants under the canopy of the eucalyptus plantation in 2012 was due to the structure of its rows, which left many empty spaces that were used by the invasive species to settle down. The number of *A. dealbata* plants decreased drastically in the next 2 years because they were cut due to the maintenance of the eucalyptus plantation. It is known that eucalyptus species impacts negatively the dynamics, composition, and structure of ecosystems (Richardson et al. 2007), but *A. dealbata* was able to successfully survive in such circumstances. The main damages attributed to eucalyptus plantations are related to soil acidification, diminution of soil organic carbon, increase of aliphaticity degree of humic substances, and increase of affinity and capacity of hydrolytic activity from soil microbial communities for forested sites (Carrasco-Letelier et al. 2004; Yelenik et al. 2004; Galatowitsch and Richardson 2005). Also, eucalyptus plantations increase water loss through the high evapotranspiration rates of these exotics trees compared with those of native flora (Le Maitre et al. 2000) and play a role in the development of soil water repellency (Ruwanza et al. 2013). However, a greater productivity has been seen in mixed rather than monospecific stands of *Eucalyptus globulus* and *Acacia mearnsii* (Forrestera et al. 2004). In addition, the positive relationship between Eucalyptus and *Acacia* species has been confirmed by the introduction of *Acacia mangium* in the understory of eucalyptus in order to increase its biomass, because of the nitrogen input brought by *A. mangium* (Laclau et al. 2008). In this framework, nitrogen-fixing Australian acacias that includes some of the most important plant invaders on a global scale (Richardson and Rejmánek 2011) have great influence on nitrogen and carbon dynamics, being able to successfully establish and grow even in resource-limited ecosystems (Funk and Vitousek 2007). In this study, *A. dealbata* overcame the light limitations and ecosystem disturbance associated with eucalyptus plantations and was able to establish in the undergrowth with relative ease. It is important to note that the planting design and the eucalyptus canopy allow greater light penetration than the native forest, where trees are distributed stochastically with a canopy of different dimensions and often laterally expanding.

The good performance of *A. dealbata* growing in shady conditions was demonstrated from early ontogenetic stages. The high GP achieved in all light levels indicates that the typical shade of Mediterranean forests is not an impediment to *A. dealbata's* establishment from seeds under its canopy. Normally, the GP of *A. dealbata* does not exceed 20% (N. Aguilera et al. Unpubl. data). Therefore, according to these authors, the values obtained in the present study indicate an germination process in accordance with the responses of *A. dealbata* seeds stored for 2 years, because they have lost the latency insofar as time elapses. In this sense, a recent study of soils invaded by *A. longifolia* (congener *A*. *dealbata*) in Portugal shows low (<12%) seed germinability (Marchante et al. 2010); but the residence time of seeds in the seed bank was not specified.

Furthermore, it is also important to note that light levels used in this study are not constant over time under the canopy of the forests, because they correspond to cloudy, partly cloudy, and sunny days. Such light levels in these ranges may fluctuate during 1 day, depending of the weather variations. Light intensities out of the canopy (full sun) can range from about 500 to 800 *μ*mol m^−2^sec^−1^ (and even reach 2000 *μ*mol m^−2^sec^−1^ at midday), so *A. dealbata* is able to respond successfully to a wide range of light availability for its establishment and normal growth. This statement is consistent with the maximum photosynthetic performance of *A. dealbata* in light intensities higher than 2000 *μ*mol m^−2^sec^−1^ (Hunt et al. 2006). In this sense, it has been suggested that the performance of this and other invasive species in wide gradients of light intensities could be an indicator of high phenotypic plasticity (Hunt et al. 2006; Funk 2008; Godoy et al. 2011). It has been considered that phenotypic plasticity is the capacity of an organism to modify its phenotypic expression in response to environmental changes (West-Eberhard 2003). Phenotypic plasticity may be an adaptive feature for plants (Sultan 1995), and this is verified when changes in phenotypic expression of functional traits in response to a particular environment enhance plant fitness (Pigliucci 2001). Thus, plasticity may increase ecological breadth of invasive species, allowing them to express advantageous phenotypes in a broader range of environments (Richards et al. 2006; Richardson and Pyšek 2006). High phenotypic plasticity has been linked to plant invasiveness because it facilitates colonization and rapid spreading over large and environmentally heterogeneous new areas (Godoy et al. 2011).

Despite the fact that *A. dealbata* can grow under really low light intensities (simulating the conditions under the canopy), this species is also able to reach high photosynthetic rates which, from a physiological point of view, suggests plastic responses to light and likely shade tolerance. In *A. mangium*, shade tolerance was associated with growth under red radiation (Yu and Ong 2003), indicating that shade tolerant behavior could be modulated for some specific length absorption from the photosynthetic active radiation spectra (PAR). In our study, high photosynthetic performance of *A. dealbata* was observed under low light intensities, reaching around 70% of PSII which indicates that most of the energy captured at low light intensity is assimilated photochemically, contributing to photosynthesis and related metabolic processes. Considering the seedlings that we tested, our results indicate that its photosynthetic machinery is highly efficient, even under the earliest ontogenetic stage and at very low light levels, which undoubtedly determines its invasiveness. Similarly, high photosynthetic efficiency was reported for others species of *Acacia* (*A. auriculiformis* and *A. mangium*), which showed high photosynthetic performance growing at different levels of the canopy (Atkin et al. 1998). These authors studied ten species of *Acacia* and observed that photosynthesis capacity depends on diurnal patterns of irradiance, suggesting that individuals able to dominate the canopy (adults) had higher photosynthetic rates that the ones reported in our study. High photosynthetic rate in *Acacia* species has been reported to be responsible for their high growth rate (Yu and Ong 2003) and has also been strongly related with their nitrogen content (Evans 1989; Evans et al. 2000).

It has been suggested that both reaction center and antenna quenching function in vivo to different extents to protect PSII from photodamage, depending on the species as well as the environmental conditions (Ivanov et al. 2008). The maintenance of an important level of intrinsic dissipation (between growth and measurement light), which was between 16% and 29% in our study, could indicate that dissipation at the reaction center level could play an important photoprotection role in shade plants, constituting a mechanism to avoid damage by excess of light. This mechanism also has been proposed as one of photoprotection against desiccation in mosses (Heber et al. 2006).

On the other hand, the low performance of PSII under high light intensities may reflect acclimation to low light intensity (Taiz and Zeiger 2006), which probably occurs with individuals growing under the canopy. To cope with the excess of energy (e.g., sun flecks that are very common under the canopy), some plants are able to develop photoprotective mechanisms to avoid damage by high irradiance. In our results, the activation of heat dissipation (increase in NPQ) under high growth light conditions suggests that during its development, at early ontogenetic stages (like seedlings that were used in this study), *A. dealbata* starts to develop mechanisms of photoprotection in order to cope with light excess (Barton 2013). This determines that adult individuals of this species have a robust photosynthetic apparatus well adapted to light, a feature that could certainly explain, at least partly, the high invasive capacity of *A. dealbata*. Increase in NPQ, which is directly related with the xanthophyll cycle (Demmig-Adams and Adams 1996), seems to be a common mechanism in *Acacia* species to cope with excess of energy. For instance, in tropical *A. crassicarpa*, the reduction of its phyllode angle to maximize the light capture, thus increasing its xanthophyll cycle activities, also plays a photoprotective role (Liu et al. 2003).

Through this research, we demonstrated that *A. dealbata* in a Mediterranean ecosystem shows an optimum photosynthetic performance, even when it grows in very low light environments. This indicates that this species, typically considered as light demanding in its native range, can become shade-tolerant in its non-native range (DeWalt et al. 2004). In general, shade tolerance has been a hotly debated and controversial issue. However, several authors agree that competition in a given environment favors plants whose form and physiology maximize their net rate of carbon gain (Givnish 1988; Walters and Reich 1999; Valladares and Niinemets 2008). The main traits associated with shade tolerance have been: lower respiration rates, higher photosynthetic rates in low light, lower leaf light compensation points and higher apparent quantum yields, higher leaf area per unit leaf dry mass, and higher whole-plant leaf fractions (leaf area ratio, leaf mass ratio) (Walters and Reich 1999; Valladares and Niinemets 2008). In this sense, the present study is the first shade tolerance approach of *A. dealbata* in its non-native range. It also provides evidence and scientific basis that suggest the usefulness of conducting deep morphological and biochemical studies in order to explain the maximization of photosynthetic carbon gain of this species in low light conditions.

In conclusion, *A. dealbata* – in the three study sites – shows clear signs that its invasion front could gradually penetrate different native and non-native forests that match the distribution range of this invasive species in the Mediterranean region of South America. Therefore, this study confirms that *A. dealbata* is a highly adaptive tree species in the Mediterranean regions of the world (Lorenzo et al. 2010; Fuentes-Ramirez et al. 2011; Richardson and Rejmánek 2011; Lazzaro et al. 2014). In invasion ecology, it is generally assumed that undisturbed forests are highly resistant to plant invasions (Martin et al. 2008). However, it has been revealed that this assumption is not strongly sustained because in temperate and tropical regions around the world, at least 139 exotic plant species are known to have invaded deeply shaded forest understories that have not undergone substantial disturbance (Martin et al. 2008). In this regard, *A. dealbata* is an another example of exotic species that has proved capable of invading forests regardless of their degree of disturbance and shade intensity. To our knowledge, this is the first study that discusses the likely shade tolerance of this invasive species in its non-native range and that provides physiological basis to understand better this particular behavior. In particular, we make an emphasis on the high photosynthetic performance that *A. dealbata* showed under very low light intensities. Finally, it should be noted that *A. dealbata*'s slower rate of invasion of native forests inwards may have lulled us into ignoring its potentially severe and long-term impacts on Mediterranean forest ecosystems of South America and probably worldwide.
